# PM_2.5_ on the London Underground

**DOI:** 10.1016/j.envint.2019.105188

**Published:** 2020-01

**Authors:** J.D. Smith, B.M. Barratt, G.W. Fuller, F.J. Kelly, M. Loxham, E. Nicolosi, M. Priestman, A.H. Tremper, D.C. Green

**Affiliations:** a MRC Centre for Environment & Health, King’s College London, UK; b NIHR Health Impact of Environmental Hazards HPRU, King’s College London, UK; c Faculty of Medicine, University of Southampton, UK; d NIHR Southampton Biomedical Research Centre, Southampton, UK

**Keywords:** PM_2.5_, Subway, Exposure, Composition, Metro

## Abstract

**Introduction:**

Despite the London Underground (LU) handling on average 2.8 million passenger journeys per day, the characteristics and potential health effects of the elevated concentrations of metal-rich PM_2.5_ found in this subway system are not well understood.

**Methods:**

Spatial monitoring campaigns were carried out to characterise the health-relevant chemical and physical properties of PM_2.5_ across the LU network, including diurnal and day-to-day variability and spatial distribution (above ground, depth below ground and subway line). Population-weighted station PM_2.5_ rankings were produced to understand the relative importance of concentrations at different stations and on different lines.

**Results:**

The PM_2.5_ mass in the LU (mean 88 μg m^−3^, median 28 μg m^−3^) was greater than at ambient background locations (mean 19 μg m^−3^, median 14 μg m^−3^) and roadside environments in central London (mean 22 μg m^−3^, median 14 μg m^−3^). Concentrations varied between lines and locations, with the deepest and shallowest submerged lines being the District (median 4 μg m^−3^) and Victoria (median 361 μg m^−3^ but up to 885 μg m^−3^). Broadly in agreement with other subway systems around the world, sampled LU PM_2.5_ comprised 47% iron oxide, 7% elemental carbon, 11% organic carbon, and 14% metallic and mineral oxides. Although a relationship between line depth and air quality inside the tube trains was evident, there were clear influences relating to the distance from cleaner outside air and the exchange with cabin air when the doors open. The passenger population-weighted exposure analysis demonstrated a method to identify stations that should be prioritised for remediation to improve air quality.

**Conclusion:**

PM_2.5_ concentrations in the LU are many times higher than in other London transport Environments. Failure to include this environment in epidemiological studies of the relationship between PM_2.5_ and health in London is therefore likely to lead to a large exposure misclassification error. Given the significant contribution of underground PM_2.5_ to daily exposure, and the differences in composition compared to urban PM_2.5_, there is a clear need for well-designed studies to better understand the health effects of underground exposure.

## Highlights

• PM_2.5_ concentrations on the London Underground are higher than ambient and other subway systems.
• Dust is generated by the wear of train components and resuspended by passing trains.
• Variability is caused by ventilation rates and is highest in long underground sections.
• The differing chemical composition of PM_2.5_ necessitates calibration of portable instruments.

## 1 Introduction

There are on average 2.8 million trips per day on the London Underground (LU) by residents and visitors to the capital, with an estimated mean journey duration of 47 min (Transport for London, 2015). Exposure to fine particles (PM_2.5_) on subway systems has been accepted as an important contributor to urban population exposure around the globe (Martins et al., 2015, Grass et al., 2010, Klepczyńska-Nyström et al., 2012) yet its potential effect on the health of the users of the system is not well understood. There are a number of clear reasons why exposure to underground railway air may be hypothesised to pose a risk to health through adverse outcomes including mortality, ischaemic heart disease, stroke, lung cancer, and chronic obstructive pulmonary disease – particulate matter (PM) concentration and PM composition (Burnett et al., 2018, Lelieveld et al., 2019).

Concentrations of airborne PM in underground railways is often several times higher than that above ground. PM_2.5_ concentrations up to 100 μg m^−3^ have been reported in subway systems in Helsinki, Los Angeles, New York, Mexico, Paris, Shanghai and Taipei (Martins et al., 2015). Concentrations in excess of 100 μg m^−3^ have also been reported in the subway systems of many cities: Barcelona (125 μg m^−3^, (Querol et al., 2012)), Stockholm (258 μg m^−3^, (Johansson and Johansson, 2003) and Seoul (129 μg m^−3^, (Sohn et al., 2008)). In London, Seaton et al. (2005) reported PM_2.5_ concentrations underground as 270–480 μg m^−3^. These particularly high PM concentrations are probably due to the age, depth, tunnel distance and limited ventilation in the LU system. Rivas et al. (2017) also measured PM_2.5_ on the LU, but as part of a series of journeys comparing exposure between transport modes, rather than focusing on the LU findings in-depth (or their spatial variation).

Many studies have measured the chemical composition of PM in the subway (Seaton et al., 2005, Adams et al., 2001, Aarnio et al., 2005, Cha et al., 2018), reporting large contributions of iron. In London, Seaton et al. (2005) found that the PM_2.5_ mass comprised approximately 67% iron oxide (Fe_2_O_3_), 1–2% quartz, and traces of other metals. In Barcelona, Querol et al. (2012) undertook a comprehensive analysis of the bulk chemical composition of PM_2.5_, the results can be summarised as 52% Fe_2_O_3_, 35% organic aerosols, 13% minerals and 6% secondary inorganic aerosols.

The size distribution of PM in subway systems is less well understood. Priest et al. (1998) were the first to measure particle size distribution in the LU and used a cascade impactor, reporting that most particles were smaller than 2.2 μm and 23% were <1 μm. Seaton et al. (2005) measured the particle number size distribution in Holland Park, Hampstead and Oxford Circus stations using a P-Trak; mean concentrations were 29,000, 14,000 and 24,000 particle cm^3^ respectively and mean particle diameters were 0.35–0.4 μm. It is difficult to compare the size distribution with above ground kerbside concentrations where the ultrafine mode is dominated by vehicle emissions peaking at 0.02–0.03 μm measured using the SMPS/APS system by Beddows et al. (2010), as this is close to the size cut off of the P-Trak. More recent measurements of particle number across a wide range of particle sizes in a railway tunnel in Stockholm (Cha et al., 2018) demonstrated that a fine fraction of PM (100–500 nm) was generated by railway-related mechanical wear while a coarse fraction (0.5–10 μm) was related to the movement of trains. This coarse fraction is known to dominate the airborne particle mass in subway systems with PM_2.5_ contributing only 27–31% in the LU (Seaton et al., 2005) compared to 73% at urban background and 66% at roadside locations in London during the same year. Previous subway studies (e.g. Aarino et al., 2005) have found that, due to the common source of PM in subway systems and the even size distribution across all particle sizes (Sitzmann et al., 1999), PM_2.5_ and PM_10_ concentrations are highly correlated.

The concentration of PM in the LU is influenced by the air drawn into the system, principally through the above ground areas of the network via tunnel entrances and through station entrances by the piston effects of the train movements as well as via active ventilation shafts. The elevated concentrations are caused by additional sources within the network, principally the wear of train consumables (e.g. wheels, collector shoes, brake blocks, motor brushes and stick lube), non-train sources (e.g. rail wear, rail grinding, ballast), station sources (e.g. escalators), and refurbishment work (which is episodic and localised) and are common to subway systems around the world (e.g. Chillrud et al., 2004, Jung et al., 2012, Querol et al., 2012). All of which are then resuspended by the train movements (Querol et al., 2012).

The microbiological contribution to aerosols on subway systems is also a major area of research, being of concern for public health and to provide bioterrorism pre-event information (Robertson et al., 2013) and is the subject of large international studies such as MetaSUB (Mason et al., 2016). Anthropogenic sources are acknowledged as major contributors to airborne bacteria at subway stations (Dybwad et al., 2012, Hwang and Park, 2014, Triado-Margarit et al., 2017) as are fungal spores (Gilleberg et al., 1998; Picco and Rodolfi, 2000).

The diverse PM sources have differing compositions: wheels and rails are steel; the collector shoes are cast iron; brake blocks vary but are typically comprised of glass fibre, metals and organic material; wheel flange stick lube is comprised of a styrene compound containing molybdenum disulphide; motor brushes are carbon. The strength and composition of these sources varies between different subway systems in different cities, for instance some use catenary systems for power supply, this leads to varying relative concentrations of metallic components (e.g. Cu) while some systems (e.g. the Métro Line 14 in Paris) use rubber wheels. Nevertheless, there is a consistent elevation of iron across subway systems, contributing approximately 30–70% of PM_2.5_ due to wear of steel components and rails (Seaton et al., 2005, Martins et al., 2016).

The principal PM sinks in the LU are the removal through the piston effects on the trains, active ventilation portals and material removed through cleaning. The amount of material available for resuspension is therefore dependent on both the deposition rate and the cleaning frequency (Moreno et al., 2014). The routine cleaning frequency in the LU is historically defined by observational reports of litter removal to reduce trackside fires resulting from sparking rather than a desire to reduce PM concentrations. More recent work has focused on reducing inhalable and respirable dust concentrations through enhanced cleaning approaches and dust suppression.

Metallic components of particulate matter are often cited as those most likely to exert health effects based on their potential to produce damaging, reactive oxygen species in biological tissues (Kelly, 2003). Indeed, in vitro studies have shown that underground PM is able to induce oxidative damage to cells including DNA oxidation/strand breakage and lipid peroxidation in spite of an induced antioxidant response (Karlsson et al., 2005, Lindbom et al., 2007, Loxham et al., 2015). However, cohort and occupational studies on acute or chronic exposure to underground PM have failed to show any consistent health effects of underground exposure beyond small changes of unclear clinical relevance. Acute exposure studies have found no effects on lung function, but small changes in T cell counts in healthy and mildly asthmatic volunteers following a 2–8 h underground exposure (Klepczynska-Nyström et al., 2010, Klepczyńska-Nyström et al., 2012). Other observations include changes in blood coagulation markers (Klepczynska-Nyström et al., 2010, Bigert et al., 2008) and increases in circulating neutrophils and monocytes (Steenhof et al., 2014). Other studies have found no association between underground PM characteristics and nasal inflammatory mediator release (Steenhof et al., 2013) or blood coagulation markers (Strak et al., 2013, Strak et al., 2013). Furthermore, no effect of chronic underground railway exposures on risk of myocardial infarction or lung cancer has been found (Bigert et al., 2007, Gustavsson et al., 2008). The lack of consistency between in vitro and real-world in vivo findings may be due to statistical underpowering of human studies, the use of unrealistically high PM concentrations in in vitro exposure studies, or the possibility that relatively simple cell culture models are less able to buffer the effects of PM compared to the complex and dynamic interactions within tissues and organ systems in vivo (Loxham and Nieuwenhuijsen, 2019). Alternatively, the mass concentration metric may overestimate inhaled PM surface area due to the relatively high density of underground PM, the predominance of metals oxides in underground PM may result in relatively poor metal bioavailability, and/or the reduced contribution of combustion-generated PM may mean that underground air has a much lower Particle Number Concentration (PNC) than might be expected from the mass concentration – each of these factors may diminish the toxic potential of underground PM.

The aim of this study was to characterise the health-relevant chemical and physical properties of PM on the LU network. PM_2.5_ was chosen as it provided the most health relevant size fraction and, due to the high correlation between PM_10_ and PM_2.5_ and the common sources of both size fractions, still offers information on the emission sources. This work included establishing the diurnal and day to day variability, and spatial distribution of PM_2.5_ as well as an improved understanding of the chemical composition.

## 2 Materials and methods

Four distinct monitoring campaigns were carried out for this research. The first (‘Exposure comparisons’) assessed day to day variation in underground concentrations compared with those above ground. The second (‘Spatial mapping’) collected particle concentration data across most of the network. The third (‘Station platform PM_2.5_: mass and chemical composition’) measured the physical and chemical characteristics of PM_2.5_ on the southbound platform at Hampstead Northern Line station, and the fourth (‘Derivation of calibration factors for optical PM mass measurement’) derived calibration factors for optical PM mass measurement, again at Hampstead station.

### 2.1 Exposure comparisons

For campaign one, test routes incorporating below ground LU and contrasting above ground environments were selected. These ran between Waterloo, and alternating destinations of Oxford Street (high traffic location) and Hyde Park (low traffic location) via the Jubilee Line. 22 repeat journeys were made on weekday mornings over a period of five months. On each journey, a TSI AM510 SidePak (TSI Inc, Shoreview, MN, USA) was used to continuously measure PM_2.5_ mass (0.1–10 μm), and a Philips Aerasense (Royal Philips NV, Amsterdam, The Netherlands) diffusion charging monitor was used to measure ultrafine particle number (10–300 μm) and also reported mean particle diameter. The averaging period of both devices was set to one-minute, and the devices were placed in a backpack with inlet tubes fed out from the top of the bag. The backpack was placed on the seat of a carriage next to the researcher during the journey on the underground and worn while walking for two hours adjacent to congested diesel traffic (Oxford Street) or in a parkland (Hyde Park). The two locations were approximately 1.5 km apart in Central London.

### 2.2 Spatial mapping

The same monitors were used for the second campaign, when a researcher rode an LU train on all lines of the network. As no GPS location tracking was possible below ground, a detailed diary indicating time of arrival and departure at each station was linked to logged concentrations. All train lines were ridden in alternating directions of travel two to five times on separate days over a three-month period, totalling approximately 31 h of sampling (89% of LU stations sampled). Measurements were linked to the transcribed time-location diary data, and then further linked to a GIS representation of the LU network created in a PostGIS database built with data from the London Data Store (Greater London Authority, 2017).

### 2.3 Station platform: Mass and chemical composition

Measurements of the physical and chemical characteristics of PM_2.5_ and TSP were made on the southbound platform at Hampstead Northern Line station. Hampstead is the deepest station on the network and was selected as a sampling location representing PM from subway sources with minimal dilution from those above ground. PM and TSP were collected onto pre-weighed quartz fibre filters (PALLFLEX, type Tissuequartz 2500QAT-UP) exchanged every four hours using 1 m^3^/h pumped samplers (Thermo Scientific Partisol 2025, Waltham, MA USA); these were subsequently weighed and analysed for mass concentration (CEN, 2014), elemental carbon (EC) and organic carbon (OC) (Quincey et al. 2009). Both these methodologies are reference procedures for ambient regulatory purposes. PM and TSP were also sampled onto mixed cellulose esters filter media (GN-4 Metricel, 0.8 μm pore) filters every four hours (Thermo Scientific Partisol 2025) and subsequently analysed for 30 elements, including Fe and other indicators of wear products in the LU using hydrofluoric acid digestion and inductively coupled plasma mass spectroscopy (Tremper et al., 2018). Twelve PM_2.5_ and 10 TSP samples were analysed. TSP, rather than PM_10_, was chosen to normalise the measured size fraction across all instruments involved in the campaign and no PM_10_ size selective inlet was available for the 4 l/min Aethalometer flow rate (data not reported here). To account for the unmeasured components, the elemental concentrations were adjusted for their associated oxides (e.g. Fe_2_O_3_) based on previous studies (Querol et al., 2012) and using widely accepted approaches used in ambient atmospheric science (Chow et al., 2015a, Chow et al., 2015b). Crustal enrichments factors (EFs) were calculated as described by Salma et al. (2007), which used average crustal rock composition (Mason and Moore, 1982) with Al as the reference element (Zoller et al., 1974).

### 2.4 Derivation of calibration factors for optical PM mass measurement

TSI AM510 SidePak (‘Sidepak’) light-scattering laser photometers were operated continuously alongside the filter samplers in both LU (Hampstead Station) and London ambient (surface) conditions in order to derive source-specific calibration factors applicable to both subway and ambient PM_2.5_. Laser photometers are typically calibrated to ’the respirable fraction of standard ISO 12103-1, A1 Test Dust’ (formerly Arizona Test Dust). As dust from different sources has different light-scattering properties, environments with dominant source types, such as the subway, require derivation of specific calibration factors (Torrey et al., 2015, Jiang et al., 2011). In each case, continuous measurements of PM_2.5_ from the Sidepak were correlated against reference PM_2.5_ mass for each of 14 four-hour mean filter exposure periods and the regression slope, calculated using the Deming regression technique (Wu and Yu, 2018) was taken as the calibration factor. A parallel sampling method was carried out at the North Kensington Automatic Urban and Rural Network (AURN) monitoring site (location 51.4750, −0.1198) using the same equipment to derive a surface background calibration factor; this is an established location representative of urban concentrations (Bigi and Harrison, 2010).

### 2.5 Population-weighted station rankings

To quantify the potential impact of high PM_2.5_ concentrations on exposure at different stations and lines, passenger ‘tap in/tap out’ data was downloaded from the London Datastore (Greater London Authority, 2017(a)) for the year 2015. This dataset records the numbers of times that automatic gates were opened by an Oyster card on an average weekday, Saturday and Sunday. The figures used in our analysis are annual entries and exits, calculated as entries plus exits, weighting weekdays by 253, Saturdays by 52 and Sundays by 59. The weighting assumed that seven bank-holidays were represented by seven Sundays, grossing up to a 364 day year, excluding bank holidays. For each station this annual entrance/exit value was divided by the mean of all stations annual entrance/exit to give a number that represented whether a station is relatively busier or quieter than the average station - this is termed the ‘passenger ranking’.

For each of the stations on the network the mean PM_2.5_ recorded while the train was at that station was then calculated (removing data collected between stations). For stations for which only one line passes through, this is typically a minute or two of data when the train paused at the station in one direction, and then again on any repeat journeys. For stations with more than one line, concentrations were averaged across the data recorded on those lines at those stations. The recorded PM_2.5_ for each station was then divided by the mean of all stations, in a similar manner as with station passenger numbers, and termed ‘PM_2.5_ ranking’. Multiplying the passenger ranking by the PM_2.5_ ranking illustrates where high concentrations are having the most impact on passenger exposure.

## 3 Results & discussion

Results from the spatial campaigns were used to highlight how the physical properties of respirable particles in underground sections of the LU contrasted with those in above ground environments, how this varied across the network, and across locations with greatest population exposure. Mass and chemical analysis then established the potential for these particles to impact on population health based on established ambient regulatory guidelines. In all cases, ambient and below ground scaling factors have been applied to PM_2.5_ mass measurements made by optical measurement devices, as described in the methods section.

### 3.1 Exposure comparisons

**Fig. 1:**
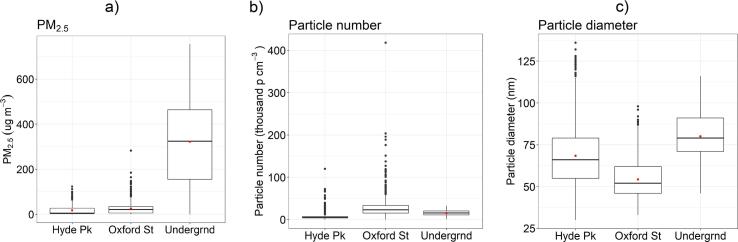
(a)–(c): Boxplot summary statistics for PM_2.5_, particle number, and particle diameter in each of the environments sampled. The lower and upper hinges correspond to the 25th and 75th percentiles, the horizontal line to the median, and the whiskers to 1.5 × the IQR (approx. 95% percentile). The red circle shows the mean. (For interpretation of the references to colour in this figure legend, the reader is referred to the web version of this article.)

Measurements taken over the 22 repeat journeys were aggregated to contrast underground, high traffic and parkland surface concentrations of PM_2.5_ mass, particle number and mean particle diameter (Fig. 1a–c).

Fig. 1(a and b) shows how PM_2.5_ mass was approximately 15 times greater in the LU (mean 302 μg m^−3^, median 318 μg m^−3^) than in surface background (mean 18 μg m^3^, median 5 μg m^−3^) and roadside environments (mean 26 μg m^−3^, median 22 μg m^−3^) in central London. While there were fewer particles measured (Fig. 1b) in the underground (mean/median: 15,070/15,790 particles cm^−3^, 77/75 nm) than the high traffic surface environment (mean/median: 26,810/22,390 particles cm^−3^, 55/53 nm), the mean particle number was many times higher than the surface background environment of Hyde Park (mean/median: 6521/5058 particles cm^−3^, 68/66 nm).

These particle number concentrations were close to those measured by Seaton et al. (2005) on the LU but the size distribution was much smaller, principally due to the lower diffusion charging measurement size cut off compared to the cascade impactor. The mean size distribution reported here (77/75 nm) was similar to the 170 nm mode reported recently by Cha et al. (2018) using a TSI Fast Mobility Particle Sizer Spectrometer (FMPS Model 3091) and Dekati Electrical Low Pressure Impactor (ELPI+).

### 3.2 Spatial mapping

The characteristics of the LU that may affect PM concentration and composition across lines are not homogenous. The age of the line, the type/model of the trains in use, the construction technique, the carriage breaking mechanisms, the passenger density and the depth of the station are all likely to be influencing factors – as they are on many underground railway systems worldwide (Harrison and Hester (Eds), 2009). For example, the Metropolitan Line opened in 1863, has large stretches of track that are above ground, a mix of shallow and deep stations when underground, was at one time used by steam trains, and is 67 km in length. In comparison the Victoria Line opened in 1968, does not have any sections that are above-ground, has deep stations, has always been electrified, and is only 21 km in length.

**Fig. 2:**
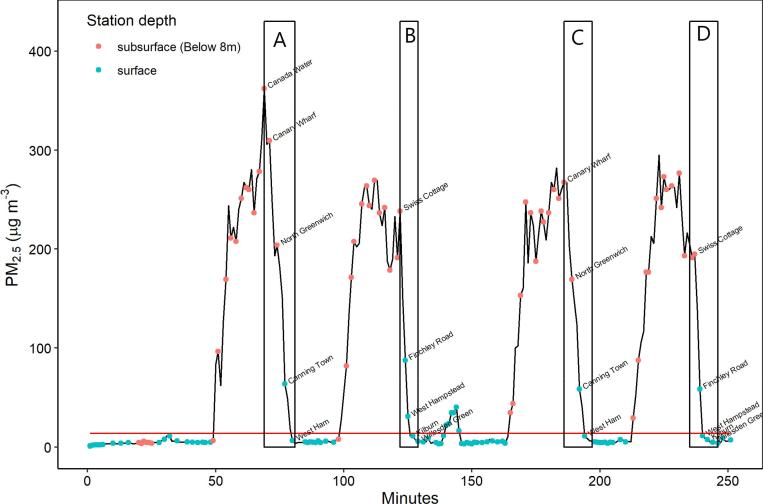
PM_2.5_ concentrations recorded on the Jubilee Line. Stations are marked with either red or blue circles depending on depth, and a background PM_2.5_ concentration taken from the ’Kensington and Chelsea – North Kensington’ background monitoring site is shown with a red horizontal line. Areas of discussion in the text are demarked A to D. (For interpretation of the references to colour in this figure legend, the reader is referred to the web version of this article.)

Fig. 2 shows how PM_2.5_ concentrations varied during a journey on the Jubilee Line. Concentrations at the ’Kensington and Chelsea - North Kensington’ background monitoring site in London, on the day of the monitoring, are shown as a red horizontal line. This site is used as an indication of ambient urban background concentrations.

PM_2.5_ concentrations during the first 50 min of the journey were similar to the London background concentrations while the train was above ground in the north section of the Jubilee Line. They then build-up inside the cabin when the train enters tunnels, before starting to fall after Canada Water, reaching background levels by the time the train reaches 0 m depth at West Ham (Boxes A and C in Fig. 2). The depths of the stations during this journey are Canada Water (18 m), Canary Wharf (18 m), North Greenwich (15 m), Canning Town (2 m), and West Ham (0 m). The first three stations are all at similar depth, yet concentrations start to fall towards levels approaching background the closer the station is to a surface station. This suggests that whilst depth is an indicator of concentrations inside the trains, distance from cleaner outside air, and its exchange with air inside the cabin when the doors are open, also effects concentrations. Taking boxed sections A and D in Fig. 2, we see a similar pattern between Swiss Cottage and Willesden Green, in that the concentrations do not immediately drop to background concentrations in the way that might be expected if depth was the sole determinant.

**Fig. 3:**
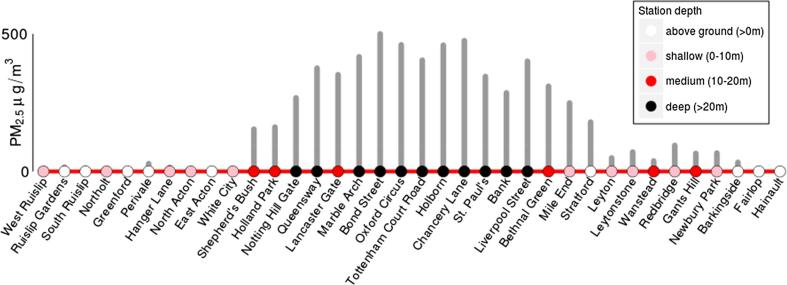
PM_2.5_ concentrations in μg m^−3^ recorded at each station of the Central Line. Station icons are colour-coded by depth (metres). (For interpretation of the references to colour in this figure legend, the reader is referred to the web version of this article.)

Further evidence of the complex relationship between depth and PM_2.5_ concentrations on the Circle Line is illustrated in Fig. 3. It is again evident that there was a relationship between line depth and PM_2.5_ concentration, with concentrations higher in the deeper parts of the line. However concentrations were again linked to distance from an above ground station; medium-depth stations flanked by deep stations (e.g. Lancaster Gate and Holland Park) had higher concentrations than medium-depth stations flanked by shallow stations (e.g. Wanstead and Gants Hill). Understanding the changes in this relationship may be important in driving improvements in air quality on the LU. Similarly, the effects of opening/closing windows, passenger numbers, and use of door screens on station platforms need further investigation.

**Fig. 4:**
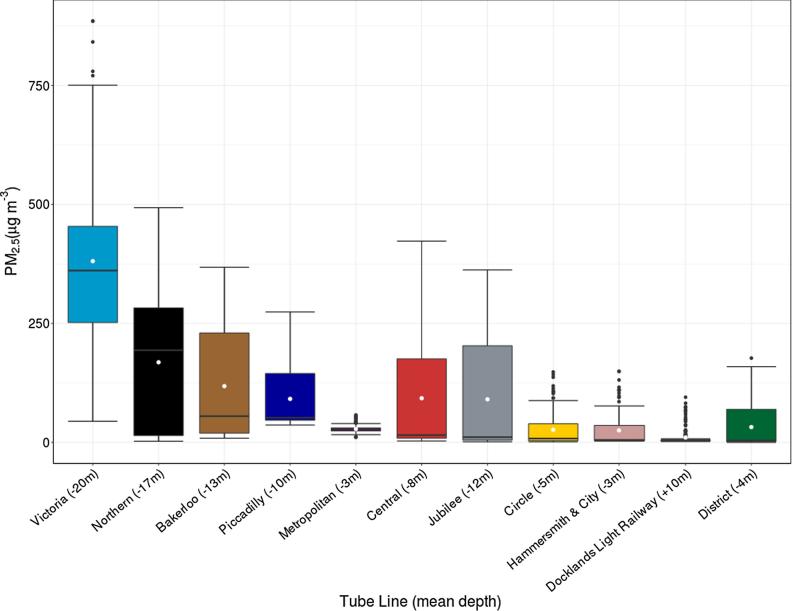
PM_2.5_ concentrations by line, ordered by median concentrations. The lower and upper hinges correspond to the 25th and 75th percentiles, the horizontal line to the median, and the whiskers to 1.5 × the IQR (approx. 95% percentile). Mean line depth shown in brackets beneath, means shown as white circles.

Summary boxplot statistics for PM_2.5_ mass for each lines of the LU are shown in Fig. 4, with the mean for each line shown as white circles.

The greatest concentrations across the network were found on the Victoria Line, which had a median PM_2.5_ concentration of 361 μg m^−3^. The other line medians, in descending order, were Northern (194 μg m^−3^), Bakerloo (55 μg m^−3^), Piccadilly (52 μg m^−3^), Metropolitan (28 μg m^−3^), Central (15 μg m^−3^), Jubilee (11 μg m^−3^), Circle (8 μg m^−3^), Hammersmith & City (5 μg m^−3^), Docklands Light Railway (4 μg m^−3^) and District (4 μg m^−3^). The highest concentrations recorded on the most polluted line (Victoria) were over 800 μg m^−3^ and were measured on the stretch of line between Pimlico and Brixton. The lowest recorded concentrations were on stretches of the Docklands Light Railway and District lines, which have large sections of line entirely above ground. SI Table 1 contains the data from Fig. 4 in tabular format. The median PM_2.5_ concentration of all data recorded on the LU was 28 μg m^−3^ (min below limit of detection, max 885 μg m^−3^).

The median concentrations recorded on trains on the Victoria (361 μg m^−3^) and Northern (194 μg m^−3^) lines were greater than any of the concentrations reported by Martins et al. (2015) from studies in Beijing, Guangzhou, Los Angeles, Mexico, New York, Seoul, Taipei, Sydney, and Barcelona. Most of the other lines on the LU, such as the Bakerloo (55 μg m^−3^), Piccadilly (52 μg m^−3^), Metropolitan (28 μg m^−3^), Central (15 μg m^−3^) and Jubilee (11 μg m^−3^) lines had similar median concentrations to those found in other studies around the world reported by Martins et al. (2015). The remaining LU lines, Circle (8 μg m^−3^), Hammersmith & City (5 μg m^−3^), Docklands Light Railway (4 μg m^−3^) and District (4 μg m^−3^) appear to be more suitably considered as above ground environments due to the large amount of time that the trains spend outside of deep tunnels.

The Victoria line has the highest PM_2.5_ concentrations, likely due to the enclosed system in which it operates. The track does not run outside or above ground and therefore the particles build-up and re-circulate within the tunnels and platforms. Ventilation is restricted to a small number of active ventilation shafts and air exchange through station entrances. The Northern line is similar, in that most of the track is underground, however near the north end of the line there are sections which run above ground, and where ventilation increases and the concentrations are consequently much lower.

The length of time that a line has been in operation might reasonably be thought to influence PM_2.5_ concentrations, on the basis that more material has been deposited over time and is then being resuspended by the movement of trains, however our data does not support this view. The Victoria line is one of the most recently constructed (1968) but has the highest median concentrations, in comparison with the Circle line, built in 1868, which has the lowest median concentrations. Other characteristics, principally ventilation, are more important.

Our data shows a great deal of variability between different lines and between locations within lines, both of which will affect a person’s exposure on a LU journey. This variability, as well as the different chemical compositions, and the need to include journey duration, make direct comparisons between journeys on the LU and other London transport modes difficult. Adams et al. (2001) collected PM_2.5_ data on 465 multi-modal journeys in London, reporting summer geometric-means of 34.5 μg m^−3^ for cycling (min 13.3 μg m^−3^, max 68.7 μg m^−3^), 39 μg m^−3^ for buses (min 7.9 μg m^−3^, max 97.4 μg m^−3^), 37.7 μg m^−3^ for cars (min 15.1 μg m^−3^, max 76.9 μg m^−3^) and 247.2 μg m^−3^ for the LU (min 105.3 μg m^−3^, max 371.2 μg m^−3^). In a similar winter measurement campaign the measurements were 20.2 μg m^−3^ for cycling (min 6.8 μg m^−3^, max 76.2 μg m^−3^), 30.9 μg m^−3^ for buses (min 5.9 μg m^−3^, max 87.3 μg m^−3^), 23.7 μg m^−3^ for cars (min 6.6 μg m^−3^, max 94.4 μg m^−3^) and 103.4 μg m^−3^ for the LU (min 12.2 μg m^−3^, max 263.5 μg m^−3^). Some journeys on the LU would likely be lower in exposure than using other forms of transport, but most would be much higher. More research is needed at other LU stations and on-board the trains to better understand the heterogeneity of exposure on and between transport networks.

### 3.3 Passenger exposures to PM_2.5_ mass

**Fig. 5:**
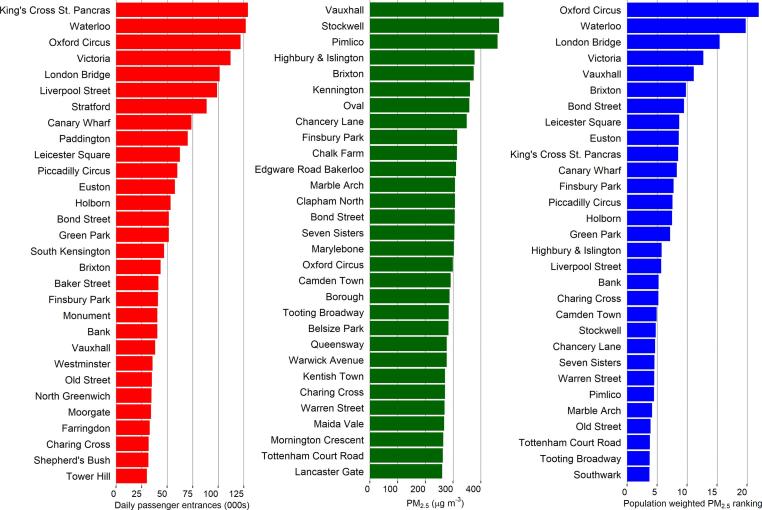
Top 30 LU stations ranked by passenger numbers (red), PM_2.5_ concentrations (green) and passenger population-weighted exposure (blue). (For interpretation of the references to colour in this figure legend, the reader is referred to the web version of this article.)

Fig. 5 shows the top 30 tube stations ranked by passenger numbers (red), mean PM_2.5_ concentrations (green) and notional passenger population-weighted exposure (mean station PM_2.5_ mass multiplied by passenger number, blue).

The passenger population-weighted exposure analysis highlights stations that could be prioritised for remediation. For example, King’s Cross St Pancras underground station was not within the top 30 stations for PM_2.5_ concentrations (shown in green in Fig. 5), but due to large passenger numbers it was the 10th highest station in terms of population-weighted exposure. In contrast, Pimlico had the 3rd highest PM_2.5_ concentrations recorded; yet due to its relatively low passenger numbers, it was 25th in the population-weighted exposure ranking. The full ranking of stations is shown in SI Fig. 1. Weighting the stations in this manner allows policymakers to consider where intervention strategies could have greatest impact.

The population of London who are making LU journeys typically spend between 17 min (0–5 years old) and 20 min (45–59 years old) on each trip they undertake, taking between 2.6 (0–5 years old) and 2.8 (17–24 years old) trips each day (Transport for London, 2017). Other age groups fall between these ranges. This means that travellers, including sensitive subgroups such as the young and the elderly, are spending between 44 and 57 min of their day in an environment where PM_2.5_ concentrations are many times higher than ambient concentrations.

Mean PM_2.5_ concentrations per station, post-calibration, are provided as a CSV file in Appendix E for use in future exposure and epidemiological research.

### 3.4 Station platform PM_2.5_: Mass and chemical composition

**Fig. 6:**
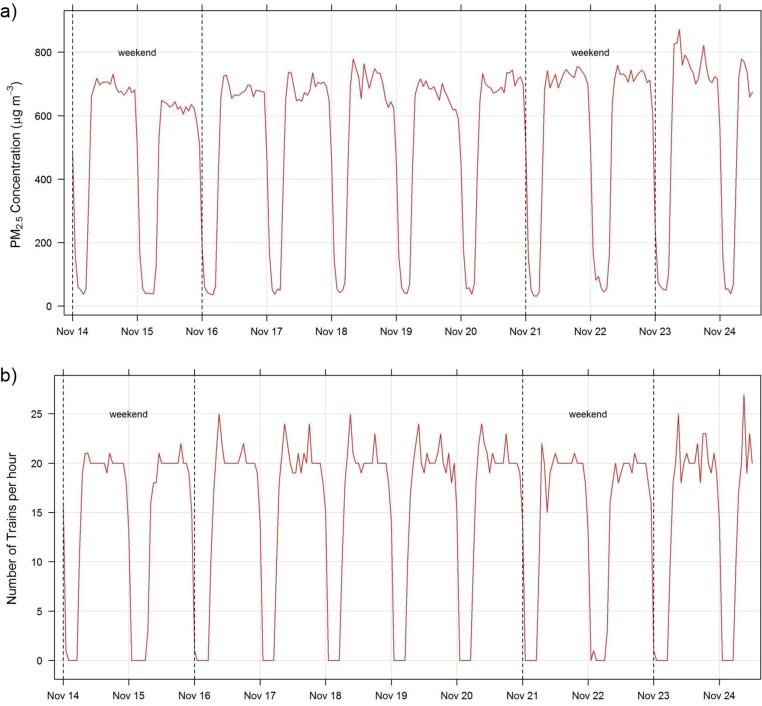
(a) High time resolution measurements of PM_2.5_ (b) The number of trains on the southbound platform at Hampstead Station.

Fig. 6 shows the measurements of PM_2.5_ mass made on the platform at Hampstead station using the Sidepak. There was a similar pattern on each day (including weekends), which was highly correlated (r^2^ = 0.96) with the number of trains passing through the platform. Minimum concentrations (mean of 97 μg m^−3^) were routinely observed between the hours of 03:00 and 06:00. Maximum concentrations (mean 658 μg m^−3^) were observed between 09:00 and midnight.

PM_2.5_ was sampled for 48 h (12 4-hour samples) and TSP was sampled for 40 h (10 4-hour samples) and filters were analysed to produce a comprehensive chemical composition breakdown; similar to Querol et al. (2012). A summary of this data is shown in Table 1; which also includes crustal enrichment factors (EFs).

**Table 1:** PM_2.5_ and TSP chemical composition and crustal enrichment factors measured on Hampstead station platform; sorted by decreasing PM_2.5_ crustal enrichment factor.

Component	PM_2.5_ (ngm^−3^)				TSP (ngm^−3^)				Crustal Enrichment Factor	
	mean	median	min	max	mean	median	min	max	PM_2.5_	TSP
Mo	1387	1653	397	2193	3041	3648	926	4329	14,836	17,360
Se	10	10	3	23	10	8	0	20	2370	1274
B	528	548	407	630	610	603	555	740	723	445
Cd	3	4	1	4	7	8	2	9	235	285
Sb	4	5	1	6	9	10	3	13	214	270
As	13	11	3	34	31	24	2	94	73	92
Li	71	91	22	99	123	142	47	168	49	46
Cr	780	915	350	1099	1249	1440	519	1705	46	39
Fe	183,648	226,160	56,551	254,680	329,864	392,174	116,443	457,063	43	41
Zn	469	437	11	2345	823	812	376	1117	43	40
Mn	2233	2790	658	3127	3748	4472	1298	5257	26	24
Cu	143	186	40	196	288	330	93	410	24	26
Pb	20	21	1	52	96	56	5	500	24	60
Cs	3	4	1	4	5	6	2	7	19	17
Ni	77	94	22	139	154	181	50	216	11	12
Nb	12	13	5	17	17	19	7	23	8	6
Co	15	19	4	20	31	36	10	44	6	6
Ca	16,991	21,204	6454	23,329	30,581	34,808	11,556	42,159	6	5
P	281	196	32	671	417	524	37	738	3	2
V	19	24	5	30	37	42	10	52	1	2
Ba	27	34	5	41	68	71	19	102	1	2
K	2460	3157	779	3550	4594	5306	1692	6293	1	1
Al	6908	8971	2080	10,538	12,946	14,944	4028	18,090	1	1
Na	1902	2090	486	3203	3506	3674	1217	4462	1	1
Sr	19	25	4	28	39	42	10	55	1	1
Ti	222	246	137	317	331	334	160	553	1	0
La	2	2	1	2	3	3	1	5	1	1
Ce	2	2	–	5	5	5	3	8	0	1
Mg	659	794	179	1099	1270	1317	369	1678	0	0

**Fig. 7:**
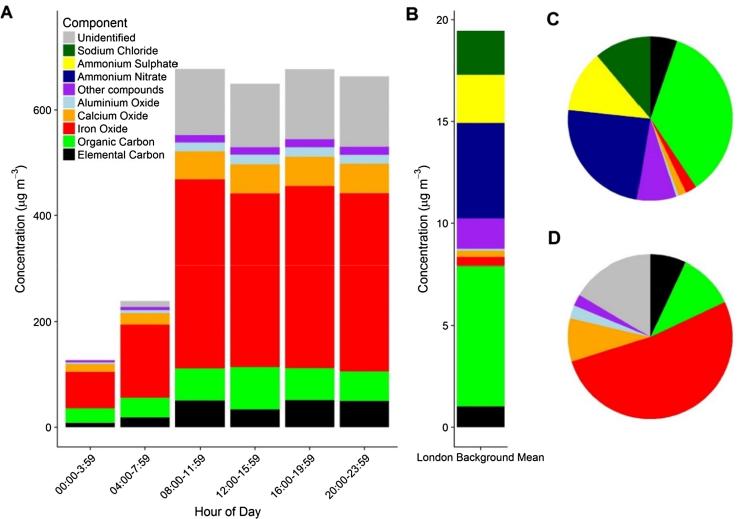
Chemical composition of PM_2.5_ as A – Hampstead Station at four-hour time resolution, B – bar chart of mean surface London background, C – pie chart for surface London background, D – pie chart for Hampstead Station.

The elements that contribute substantially to the PM_2.5_ mass are shown in Fig. 7 where data are expressed relative to the independently measured total mass. PM_2.5_ was found to contain 47% Fe_2_O_3_ while the remaining mass was made up of elemental carbon (32 μg m^−3^, 7%), organic carbon (51 μg m^−3^, 11%) as well as metallic and mineral oxides (14%). It was not possible to measure all the chemical components, as the techniques used were not sensitive to certain elements such as silicon and oxygen and 21% remained unidentified. Based on the crustal abundance of Si to Al (Mason and Moore, 1982), accounting for SiO_2_ in resuspended mineral material would reduce the missing mass to <10%.

The Fe_2_O_3_ contribution to PM_2.5_ measured in this study was consistent with other studies on the LU (Sitzmann et al., 1999, Nieuwenhuijsen et al., 2007) but lower than the 67% reported by Seaton et al. (2005) at the same location (by transmission electron microscopy). The bulk chemical composition of PM_2.5_ measured at Hampstead station was clearly very different to surface measurements. At a typical surface London background location, organic carbon is the most abundant source (6.8 μg m^−3^, 35%) from local and distant sources followed by secondary inorganic aerosols, (ammonium nitrate (4.7 μg m^−3^, 24%) and ammonium sulfate (2.4 μg m^−3^, 12%)), marine aerosol components (sodium chloride (2.2 μg m^−3^, 7%)) and direct combustion emissions (elemental carbon (1.0 μg m^−3^, 5%)) (Bohnenstengel et al., 2014).

The diurnal variation in PM_2.5_ chemical composition was restricted to the four hour time resolution afforded by the filters but it is clear that it reflected the higher time resolution data recorded by the Sidepak, shown in Fig. 6. The lowest concentrations measured by the Sidepak were not reached in the minimum chemical composition data (00:00–03:59) as concentrations were still falling following the last train at 01:00. The next sampling period (4:00:7:59) encompasses the first trains from 05:00 until peak service (approx. 20 trains per hour) at 07:30. The composition, relative to the PM_2.5_ mass, was constant to all of the major contributors except the unidentified component, which as discussed likely relates to the unmeasured SiO_2_ and mineral composition.

EC contributed 7% to PM_2.5_ in the LU, this was similar to Helsinki, Berlin and Budapest (Aarnio et al., 2005; Fromme et al., 1998; Salma et al., 2007) but higher than the 1% reported in Barcelona (Querol et al., 2012). In Helsinki, these elevated concentrations were attributed to the ingress of polluted air from outside or the use of diesel trains for maintenance work; this is not expected to be the cause of the elevated concentrations in London. Instead, other sources of carbon material, such as the wheel flange lubricating sticks (used to reduced noise and wear on curved sections of track) and carbon motor brushes (found in the traction motor, compressor and alternator in the older train stock) are the likely source as very few diesel engines are used in the LU. Any influence of outside air is minimal as the measurement location was distant from any above ground vehicle sources.

The use of many of the metallic elements in a range of applications in the LU, such as anti-friction materials (Erdemir 2001) and steel alloys (Seetharaman 2005) make the identification of specific sources very challenging. Enrichment factors (EFs) are widely used to identify anthropogenic sources (EF > 1), although their accuracy in ambient studies is questioned due the geological variability as well as physical and chemical weathering processes (Reimann and Caritat, 2000). Here, Al was used as a tracer for crustal material as in previous studies and the results are shown in Table 1 and are mostly consistent with those studies (Salma et al., 2007, Querol et al., 2012). The difference in the enrichment factors between PM_2.5_ and TSP can also be used to identify potential sources or highlight differences between sources.

Many of the elements used in anti-friction materials were substantially enriched in the LU. Mo, Se and B had the highest PM_2.5_ EFs (17360, 1274 and 445 respectively), while Li was less enriched (49). Similar to graphite, Mo, Se and B are commonly used in dry lubricant material as they form lamellar structures (e.g. MoS_2_, MoSe_2_, BN) and hence reduce friction; Se, B and Li compounds are also used as additives to oils and greases (Erdemir, 2001). The TSP EF of Mo was greater than that in PM_2.5_ showing that it was more enriched in the coarse PM (relative to Al), the opposite was true of Se and B while Li was similarly enriched in both fractions. This indicates that the source of Mo generates particles larger than the crustal source, the source of Se and B generates particles smaller than the crustal source while Li source generates particles with a similar size distribution. Cd and Sb have PM_2.5_ EFs of 235 and 214 respectively, They are accepted constituents of brake pads having been used as tracers in previous studies (Grigoratos and Martini, 2015) and their EFs were larger in TSP than in PM_2.5_. The As enrichment may have been due to its presence as an impurity in brakes or other metallic components (Querol et al., 2012); the similar ratio of the As EFs in PM_2.5_ and TSP to those of Cd and Sb support this. However, elevated concentrations of As have also been found on surface railway lines (Smith et al., 2006, Staszewski et al., 2015) and associated with its use in herbicides, wood preservatives and pesticides and it is possible As may come from its historic use in some of these applications. Cr, Fe, Zn, Mn and Cu are all components of steel used in the wheel, rail and electrification system (Seetharaman, 2005). Pb was also notably enhanced but, unlike other elements, the TSP EF was much larger than the PM_2.5_ EF; the same was true in Barcelona (Querol et al., 2012) but not in Budapest (Salma et al., 2007). In London, this may indicate a different Pb source such as its use in paint as identified close to subway infrastructure in New York (Caravanos et al., 2006).

Pb, Cd, Ni and As are regulated by EU through the Air Quality Directive (2008/50/EC) and 4th Air Quality Daughter Directive (2004/107/EC) due to their accepted health impacts; Pb has an EU limit value, an annual mean, of 50 ng m^−3^ while Cd, Ni and As have target values of 5 ng m^−3^, 20 ng m^−3^ and 6 ng m^−3^ (also annual means) respectively. All are generally found in very low concentrations in PM_10_ above ground - central London surface concentrations of Pb, Cd, Ni and As during 2016 were 8, 0.1, 0.9 and 0.9 ng m^−3^ respectively. In contrast, concentrations measured in PM_2.5_ at Hampstead Station 20, 3, 77 and 13 ng m^−3^ respectively for PM_2.5_ and 96, 7, 154 and 31 ng m^−3^ respectively for TSP. The PM_2.5_ concentrations underestimate the short-term exposure to PM_10_ as they only capture a subset of the larger particle fraction and include a night-time, low concentration period. If these measured concentrations are representative of the whole year, both Ni and As would exceed the annual mean target value; As by a factor of 2, and Ni by a factor of 3 before any account has been made for the difference in concentration between PM_10_ and PM_2.5_.

Given the near ubiquity of underground railway systems in the world’s largest cities, and the high airborne concentrations of PM found across networks (Nieuwenhuijsen et al. 2007), it is perhaps surprising that relatively little is known about the potential health-related effects of exposures in such environments (Salma, 2009). Permanent exposure to typical urban PM at these concentrations would be expected to have impacts on health, especially given that the exposure-response function at these concentrations of PM_2.5_ appears to be steeper than previously thought when the UK’s Committee on the Medical Effects of Air Pollution (COMEAP) adopted a relative risk of mortality value of 1.06 per 10 μg/m^3^ PM_2.5_ (COMEAP, 2009), in line with findings from a large cohort study published in 2012 (Pope et al., 2002). The reason for this increased steepness is that the use of cigarette smoking in deriving exposure-response functions in earlier studies (Burnett et al., 2014) has been replaced by relatively recent ambient PM exposure studies performed in more heavily polluted cities (Burnett et al., 2018). However, this change also suggests that PM exposure-response functions may differ by PM source/composition, which has potential implications for environments where PM is chemically distinct from that found in surface environments (Li et al., 2019). The transition-metal rich nature of underground PM appears to endow it with a considerable ROS-generating capacity, potentially deriving from either wear of wheels and rails or brake components (Loxham et al., 2013, Moreno et al., 2017), and this may be expected to present increased risks to health given the role of ROS in PM toxicity (Kelly and Fussell, 2015). It should be noted that the underground particle size distribution measured in this study showed median particle diameters were larger in the LU than above ground, which will reduce the surface area to mass ratio as well as changing where particles deposit relative to typical urban PM_2.5_. Furthermore, other studies (Karlsson et al., 2005; Moreno et al., 2017) report lower solubility of PM components and hence bioavailability. Both size and solubility may act to reduce PM toxicity (COMEAP, 2019), although this latter aspect may also present concerns regarding chronic particle exposure and clearance of insoluble PM. Studies of acute exposure on the platform in the Stockholm underground, where PM_2.5_ exposure was several times lower than in the present study, found no effect on lung function, although did note small effects of unknown clinical significance suggesting increased propensity towards blood coagulation (Klepczynska-Nyström et al., 2010, Klepczyńska-Nyström et al., 2012). However, reflecting chronic exposure, no increased risk of lung cancer (Bigert et al., 2007) or myocardial infarction (Gustavsson et al., 2008) was found in underground train drivers, and a study in the New York subway similarly did not find consistent evidence of raised biomarkers (Grass et al., 2010). However, questions remain. Given the potential for ultrafine PM to enter the systemic circulation (Miller et al., 2017), there is a need to understand the effects of chronic exposure to this specific type of PM, which may only manifest in retirement. In particular, there is little understanding of the effects of particles reaching the brain (Maher et al., 2016), which may be relevant to underground PM exposures given the potential role of metals and oxidative stress in Alzheimer’s disease (Cheignon et al., 2018), and emerging associations between chronic PM exposure and dementia (Jung et al., 2015). Furthermore, there are likely to be effects of PM which extend beyond the relatively narrow range of endpoints studied this far, and these may also vary by PM composition (Huff et al., 2019). Finally, for commuters, it is important to understand the effects of acute exposure to such elevated concentrations of PM_2.5_, which may contribute to a large portion of an individual’s inhalation of specific PM components and overall PM mass concentration (Rivas et al., 2017), and which may differ depending on PM composition (Achilleos et al., 2017).

### 3.5 Calibration factors for optical PM mass measurement

Scaling factors of 0.44 for ambient PM_2.5_ concentrations, and 1.82 for LU concentrations were calculated using the methods described in 2.4 (See SI Figs. 3 and 4 for correlation plots and R^2^ values). The four-fold difference in above and below ground calibration factors highlights the importance of correctly applied scaling when using optical PM mass measurement devices in exposure studies.

PM_2.5_ concentrations recorded by the Sidepak during the spatial campaigns (‘Exposure comparisons’ and ‘Spatial mapping’) were multiplied by the derived calibration factors and are reflected in results 3.2, 3.3. In recognition that the LU air comprises a mix of below and above ground particulate sources, these factors were proportionally applied (Eq. (1), illustrated with simulated data in Appendix D, Supplementary Fig. 2.

(1) $${\left[{\text{lu}}_{\text{true}}\right]}_{\text{i}}=\left({\left[\text{lu}\right]}_{\text{i}}-{\left[\text{sb}\right]}_{\text{i}}/{\text{k}}_{\text{s}}\right)*{\text{k}}_{\text{u}}+{\left[\text{sb}\right]}_{\text{I}}$$

where lu_true_ is the calibrated concentration at time i, lu is the measured concentration, sb is the paired measured concentration from a surface background monitoring site, k_u_ is the derived underground calibration factor (1.82), and k_s_ is the surface background calibration factor (0.44).

When above ground (defined as when recorded concentrations were equal to or less than concentrations recorded at the surface background monitoring site), only the surface background factor of 0.44 was applied.

By calculating bespoke scaling factors for the optical PM_2.5_ mass monitor, we provide a robust calibration approach for their use in other studies of underground subway environments.

## 4 Conclusions

Concentrations of PM_2.5_ on the LU were many times greater than in other London transport environments, and greater than on other subway systems around the world. The high concentrations experienced in the LU were most probably due to age, depth, tunnel distance and limited ventilation. This was confirmed by the relationship between depth and air quality, however, there were clear influences relating to the distance between the measurement point and the nearest overground section of line.

Due to large differences in calibration factors, care should be taken to ensure optical particle counting measurements are calibrated to subway environments. Previous studies using these instruments with ambient calibration factors may have underestimated mass concentrations by up to a factor of four.

Total PM_2.5_ mass was found to vary between lines and locations. The line with the lowest median recorded concentrations was the District line (4 μg m^−3^, mean 32 μg m^−3^), where values are can be lower than those measured at ambient London background. The highest median concentration was recorded on the Victoria Line (361 μg m^−3^, mean 381 μg m^−3^), around 16 times higher than roadside measurements.

The PM_2.5_ sampled comprised of 47% iron oxide, 7% elemental carbon, 11% organic carbon, and 14% metallic and mineral oxides; broadly in agreement with other subway systems around the world.

The unusual composition of underground PM_2.5_, and its airborne concentrations, implies a need for studies to investigate potential effects on health, of which there are few. Those published to date are exclusively in networks with markedly lower PM_2.5_ concentrations than that in London and tend to use a small sample size and relatively healthy subjects. Knowledge gaps should be addressed by sufficiently powered acute and chronic exposure studies, utilising a wider range of endpoints and attempting to study cross sections of the population, including potentially vulnerable subgroups.

To prioritise remedial action to lower passenger exposure in subway systems around the world, a method that combines measured concentrations and passenger numbers from smartcard data or similar was demonstrated. Remedial actions might include; regular cleaning of the tunnels, platform screen doors to shield waiting passengers from the particles being resuspended as trains arrive and depart stations, and trials of ventilation systems and settings to better protect passengers inside the trains.

Failure to include this transport environment in epidemiological studies into the relationship between PM_2.5_ and health in London is likely to lead to exposure misclassification error, potentially biasing associations toward the null.

## Declaration of Competing Interest

The authors declare that they have no known competing financial interests or personal relationships that could have appeared to influence the work reported in this paper.
