# Supplementary material for: PM2.5 on the London Underground
Source: Environ Int. 2020 Jan;134:105188. doi: 10.1016/j.envint.2019.105188 (PMC6902242; doi:10.1016/j.envint.2019.105188)
Supplement: Supplementary data 1 [file mmc1.docx]

**Supplementary Information: PM_2.5_ on the London Underground**

J.D. Smith^1^, B.M. Barratt^1,2^, G. Fuller^1^, F.J. Kelly ^1,2^, M. Loxham^3,4^, E. Nicolosi^1^, M. Priestman^1^, A.H. Tremper^1^ and D.C. Green^1^

^1^MRC Centre for Environment & Health, King’s College London, UK

2 NIHR Health Impact of Environmental Hazards HPRU, King’s College London, UK

3Faculty of Medicine, University of Southampton, UK

| **Line** | **Min** | **Mean** | **Median** | **Max** |
| --- | --- | --- | --- | --- |
| Victoria | 45 | 381 | 361 | 885 |
| Northern | 3 | 169 | 194 | 493 |
| Bakerloo | 9 | 118 | 55 | 368 |
| Piccadilly | 37 | 92 | 52 | 274 |
| Metropolitan | 11 | 28 | 28 | 58 |
| Central | 3 | 93 | 15 | 423 |
| Jubilee | 1 | 91 | 11 | 362 |
| Circle | 1 | 27 | 8 | 148 |
| Hammersmith & City | 3 | 25 | 5 | 149 |
| Docklands Light Railway | 2 | 10 | 4 | 95 |
| District | < limit of detection | 32 | 4 | 177 |
| **All lines** | **< limit of detection** | **88** | **28** | **885** |

SI Table 1: Tabular summary of data presented in Figure 4 (PM_2.5_ concentrations, by line, recorded on the London Underground)

| << Provided as an accompanying file >> | SI Figure 1: London Underground stations ranked by passenger numbers (red), PM_2.5_ concentrations (green) and population-weighted exposure (blue). |
| --- | --- |

| 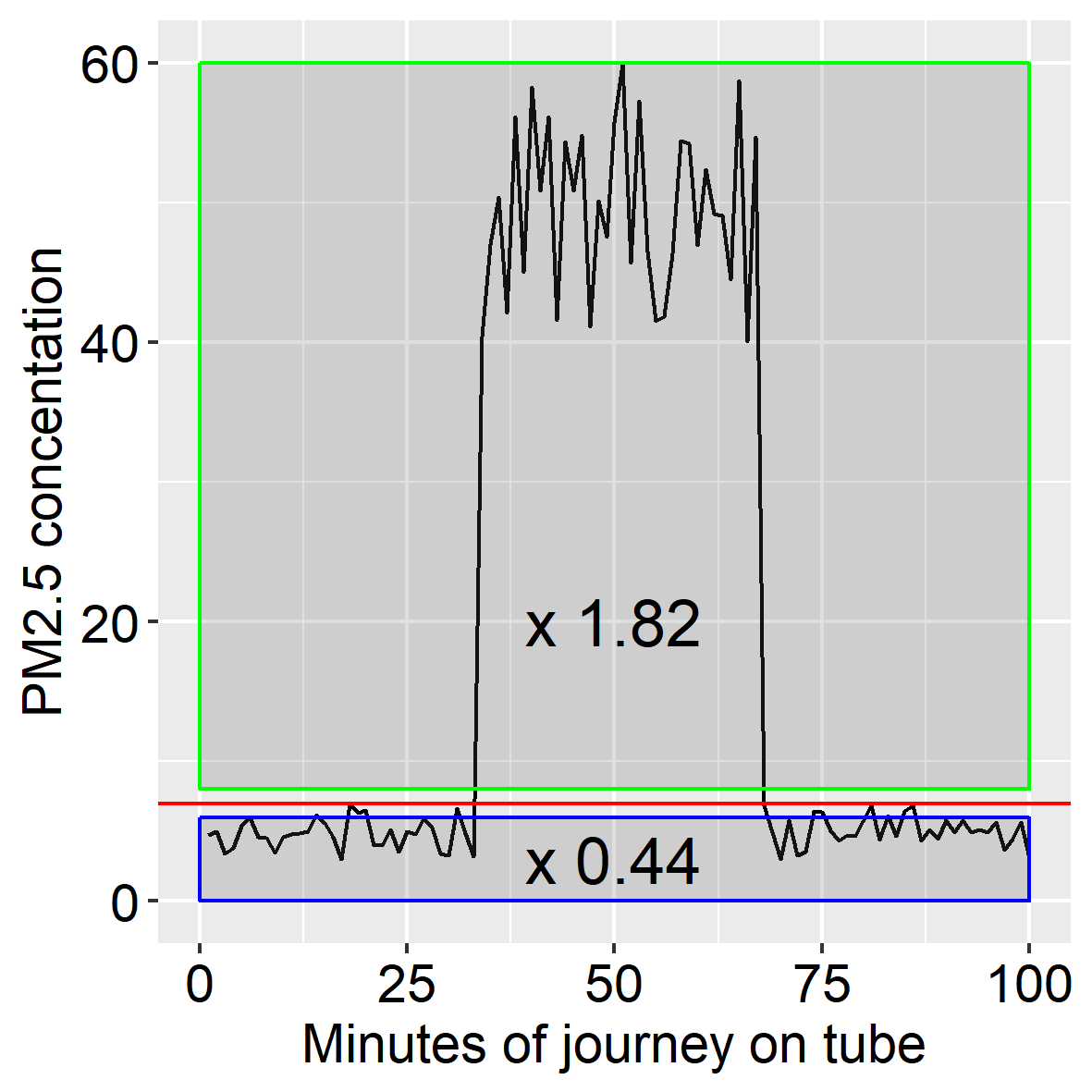 |
| --- |
| SI Figure 2: Diagram illustrating how scaling factors were applied to PM_2.5_ data collected by the TSI Sidepak when used on the London Underground. Ambient air highlighted by a bluebox, underground air by a greenbox. |

| 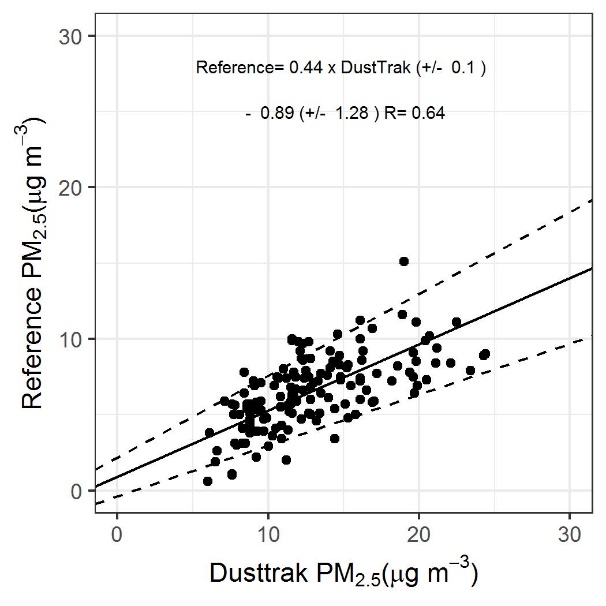 | 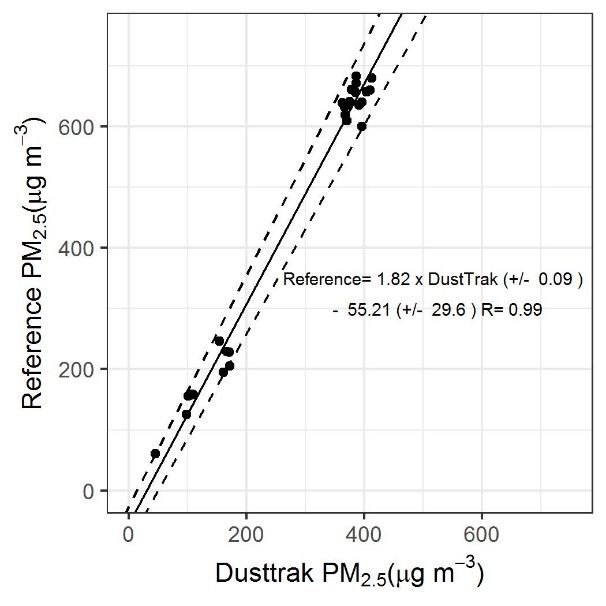 |
| --- | --- |
| SI Figure 3: PM_2.5_ measurements from a TSI Sidepak v. reference measurements, in a London outdoor environment. | SI Figure 4: PM_2.5_ measurements from a TSI Sidepak v. reference measurements, on the platform of Hampstead Station of the LU. |
|  |  |

<< LINK TO CSV FILE >>

SI Dataset 1: Mean recorded PM_2.5_ concentrations per station
