# Supplementary figures and images for: PM2.5 on the London Underground
Source: Environ Int. 2020 Jan;134:105188. doi: 10.1016/j.envint.2019.105188 (PMC6902242; doi:10.1016/j.envint.2019.105188)

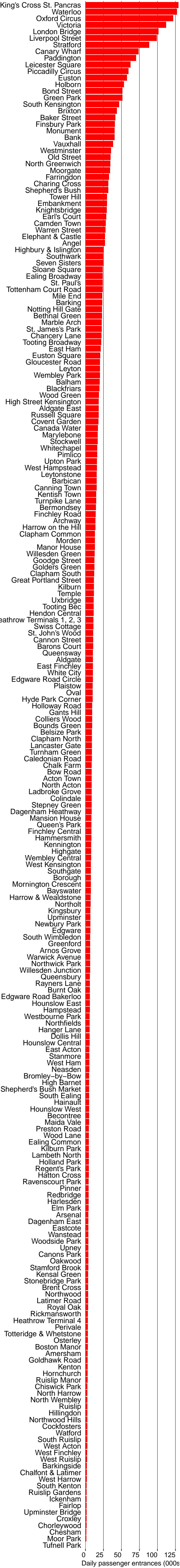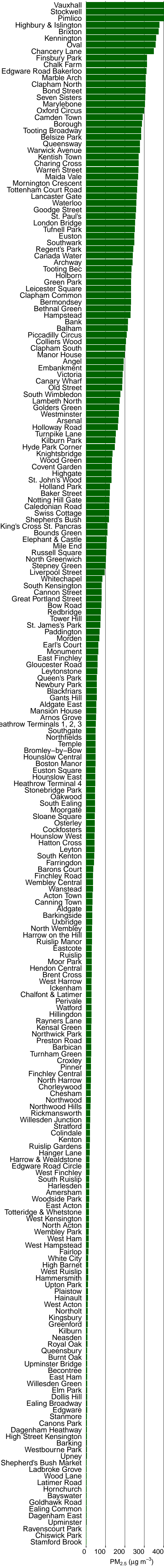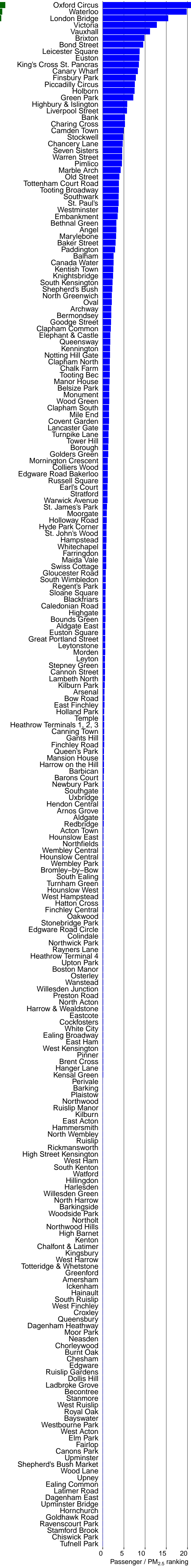

Supplement: Supplementary data 3 [file mmc3.pdf]
